# Setting reasonable goals for kidney transplant referral among dialysis facilities

**DOI:** 10.1186/s12882-024-03671-2

**Published:** 2024-07-24

**Authors:** Jessica L. Harding, Meredith A. Dixon, Mengyu Di, Julien Hogan, Stephen O. Pastan, Rachel E. Patzer

**Affiliations:** 1 Department of Epidemiology, Rollins School of Public Health, Emory University, Atlanta, GA USA; 2 Department of Surgery, Emory University School of Medicine, Atlanta, GA 30322 USA; 3 Department of Medicine, Emory University School of Medicine, Atlanta, GA 30322 USA; 4 Health Services Research Center, Emory University School of Medicine, Atlanta, GA USA; 5 Regenstrief Institute, Indianapolis, IN USA; 6 Division of Pediatric Nephrology Université Paris, Cité | Hôpital Robert Debré, APHP, Paris, France; 7 Department of Surgery, Indiana University School of Medicine, Indianapolis, IN USA

**Keywords:** Kidney transplantation, End-stage kidney disease, Referral

## Abstract

**Background:**

Determining whether a patient is eligible for kidney transplantation is complex. In this study, we estimate what proportion of patients with end-stage kidney disease (ESKD) might have been suitable candidates for kidney transplantation but were not referred.

**Methods:**

We identified 43,952 people initiating dialysis for kidney failure between 2012 and 2017 in the states of Georgia, North Carolina, or South Carolina from the United States Renal Data System and linked to the Early-Steps to Transplant Access Registry to obtain data on referral and waitlisting up until December 2020. We identified ‘good transplant candidates’ as those who were waitlisted within 2-years of referral, among all patients referred within 1-year of dialysis initiation. Using propensity score cut-offs, logistic regression, and area under the curve (AUC), we then estimated the proportion of individuals who may have been good transplant candidates, but were not referred.

**Results:**

Overall, 42.6% of incident dialysis patients were referred within one year and among them, 32.9% were waitlisted within 2 years of referral. Our model had reasonably good discrimination for identifying good transplant candidates with an AUC of 0.70 (95%CI 0.69–0.71), sensitivity of 0.68 and specificity of 0.61. Overall, 25% of individuals not referred for transplant may have been ‘good’ transplant candidates. Adding these patients to the existing 18,725 referred patients would increase the proportion of incident ESKD patients being referred within one year from 42.6% to 57.2% (a ~ 14.6% increase).

**Conclusions:**

In this study, we show that a significant proportion of potentially good transplant candidates are not being referred for transplant. A ~ 14% increase in the proportion of patients being referred from dialysis facilities is both a meaningful and realistic goal and could lead to more qualified patients being referred and subsequently waitlisted for a lifesaving transplant.

**Supplementary Information:**

The online version contains supplementary material available at 10.1186/s12882-024-03671-2.

## Background

Determining whether a patient is eligible for kidney transplantation is complex. Well documented disparities in transplant referral [1], waitlisting [2], and transplant rates among waitlisted patients [3] by non-medical factors such as race, sex, and socioeconomic status suggest there is ample opportunity to improve equity in access to transplantation [4]. However, the exact proportion of patients who are medically eligible for kidney transplantation remains unknown as not all patients with end-stage kidney disease (ESKD) undergo evaluation by a transplant program. While a number of national initiatives and quality measures have been introduced in the last few years targeting improved access to transplant waitlisting [5], there are no national benchmarks to improve access to referral for medical evaluation at a transplant center, a necessary step for eventual placement on the national waitlist. The purpose of this study is to determine what proportion of non-referred ESKD patients might have been suitable candidates for kidney transplantation.

## Methods

### Study population

**Fig. 1:**
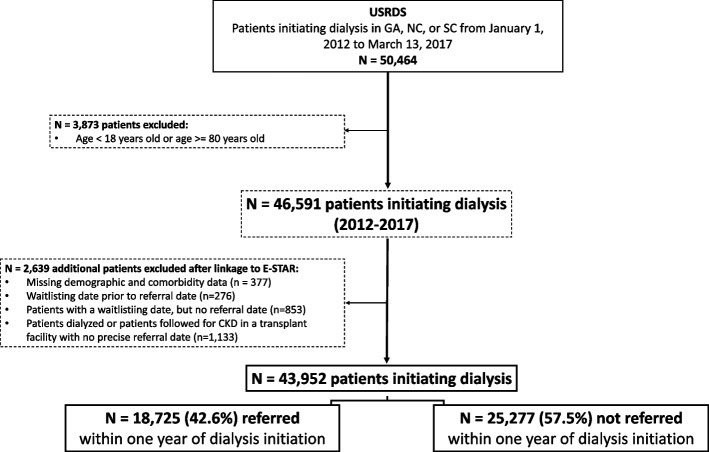
Flowchart of study population, incident dialysis patients from GA, SC, NC, 2012–2017. *Abbreviations*: *CKD* chronic kidney disease, *E-STAR* Early Steps to Transplant Access Registry, *GA* Georgia, *NC* North Carolina, *SC* South Carolina, *USRDS* United States Renal Data System

We identified all patients aged 18–80 years initiating dialysis from the United States Renal Data System (USRDS) [6], a national registry of all adults receiving kidney replacement therapy (KRT; either in the form of dialysis or transplant) for chronic kidney failure. We restricted our study sample to adults initiating dialysis Network 6 (Georgia (GA), North Carolina (NC), and South Carolina (SC)) between January 1, 2012 and March 13, 2017, and followed until December 2020. We restricted our cohort to 2012–2017 to ensure all patients had a minimum of 3-years of follow-up. We included patients who were pre-emptively referred or waitlisted (*i.e.,* prior to dialysis initiation), and excluded patients from transplant facilities with missing referral dates. As the goal of this research is around setting reasonable goals of referral for dialysis facilities, we excluded all patients who received a transplant as their first KRT modality (i.e. pre-emptively transplanted patients). Our final study sample included 43,952 adults (Fig. 1). This study adheres to the STROBE guidelines for observational studies (Table S1).

### Study outcomes – referral and waitlisting

Outcome data were obtained by linking individuals in the USRDS cohort to patient-level referral data obtained from the Early Steps to Transplant Access Registry (E-STAR), [1, 7] a voluntary registry of transplant referral and evaluation data collected from all (i.e., 100% capture) nine adult transplant centers in end-stage renal disease (ESRD) Network 6. Waitlisting data was obtained from USRDS. All individuals were followed from dialysis start date and followed through March 13, 2020 to allow for at least one year of follow-up to referral and at least two years of follow-up to waitlisting thereafter for all individuals. Two years was chosen as the time cut-off for waitlisting based on examination of the cumulative incidence of waitlisting that demonstrated that for the majority of patients who were waitlisted, they were waitlisted within 2-years of referral (see Figure S1). Specifically, approximately 29% of patients were waitlisted within 24 months, as compared with 36% (cumulatively) at 60 months (from time of referral). This was balanced with a smaller proportion of deaths occurring in this window (i.e., ~ 12% at 24 months vs. 29% at 60 months). For individuals who were pre-emptively referred or waitlisted (i.e., prior to dialysis initiation), follow-up time was calculated as 1-day. Referral was defined as date of first referral within 1-year of dialysis initiation. Waitlisting was defined as date of waitlisting within 2-years of referral date.

### Patient-level covariates

Patient-level characteristics, as recorded in USRDS at time of dialysis start, were ascertained from the Centers for Medicare and Medicaid (CMS) form 2728. Key variables of interest included attributed cause of ESKD (diabetes, hypertension, glomerulonephritis, other), age (categorized as 18–29, 30–39, 40–49, 50–59, 60–69, 70 + years in models)), sex (men or women), race and ethnicity (non-Hispanic White, non-Hispanic Black, Hispanic, and ‘other,’ where other is made up of Middle Eastern, American Indian or Alaskan Native, Asian, Indian, Pacific Islander, and multi-racial), and dialysis type (hemodialysis, continuous ambulatory peritoneal dialysis (CAPD), continuous cycling peritoneal dialysis (CCPD) or other). Other variables of interest collected on the CMS2728 form included access to pre-kidney failure nephrology care (yes, no), comorbidities (tobacco use, congestive heart failure, diabetes, hypertension, atherosclerotic heart disease, other cardiac disease, cerebrovascular disease, peripheral vascular disease, cancer, chronic obstructive pulmonary disease (COPD), and obesity defined as body mass index (BMI) ≥ 35 kg/m^2^). Insurance status was defined as no insurance, Medicaid, Medicare, employer, or other. For health insurance status, where patients indicated they had > 1 insurance provider, we categorized them using a hierarchy of employer, Medicaid, Medicare, and other. In the US, Medicare is provided to all adults ≥ 65 years, individuals with disability, or individuals receiving treatment for kidney failure. Medicaid is provided to all individuals defined as low-income based on state-specific thresholds. For all non-primary variables, excluding pre-kidney failure nephrology care, < 5% of data were missing. For pre-kidney failure nephrology care, 13.1% of data were missing.

### Statistical analysis

#### Defining a ‘good’ transplant candidate

To estimate the proportion of patients with kidney failure not referred within one year who may have been good transplant candidates, we first defined ‘good transplant candidates’ as patients waitlisted within two years of referral date among those referred within 1-year from dialysis initiation.

#### Propensity Score (PS) development

To estimate the proportion of non-referred patients potentially eligible for referral the following stepwise process was used:

First, in order to identify ‘good’ transplant candidates, we performed a multivariate logistic regression in our cohort of patients referred within one year of dialysis initiation, using waitlisting within two years as the outcome of interest. We estimated the predicted probability (*i.e.,* propensity score) of being waitlisted within two years of referral versus not being waitlisted within two years of referral by introducing explanatory clinical characteristics (age, attributed cause of kidney failure, and all patient-level comorbidities) based on a priori clinical knowledge. Type of insurance coverage, sex, and race/ethnicity were not included in the model since we only wanted to include factors that *should* be used to determine patient suitability for transplant in an ideal situation. A complete case approach was used as < 5% of data was missing across all variables included in models. The final model was used to estimate a PS for each patient.

Second, we evaluated the model's ability to distinguish between patients who were waitlisted within 2 years of referral and those who were not using the Area Under the Receiver Operating Characteristic curve (AUROC). To select the optimal PS cut-off for our model, we identified the threshold corresponding to the point closest to the top-left corner of the AUROC curve. This point balances sensitivity and specificity, providing an effective threshold for the model to accurately differentiate between waitlisted and non-waitlisted patients [8]. 

Third, we applied this PS logistic regression model to our non-referred cohort and selected the patients with a waitlisting probability within the above-mentioned PS cut-off (*i.e.,* patients with a high predicted probability of waitlisting). We compared the clinical and demographic characteristics of these non-referred patients with those of referred patients to assess non-medical factors that might explain the remaining observed differences.

### Sensitivity analyses

Given known disparities in who is and is not referred [1, 7], we also estimated PS for good transplant candidates among the entire dialysis cohort (vs. only among those referred in primary analysis). The same approach was taken as described above with some exceptions. Given all dialysis patients include those who were not referred, we no longer had a requirement of referral within 1-year plus waitlisted within 2 years of referral to define a good transplant candidate. Instead, for the total population we defined a good transplant candidate as someone who was waitlisted within 3 years of dialysis initiation. Three years was chosen to match primary analyses which included 1-year of referral plus 2-years of waitlisting. Furthermore, because referral was no longer a requirement to be a ‘good transplant candidate’, we did not define our referred and non-referred cohorts as referred (or not) within 1-year of dialysis initiation, thus sample populations are slightly different to those in primary analysis. The PS cut-off chosen to define a good transplant candidate in sensitivity analysis was similarly defined as above using AUROC curves.

## Results

### Baseline characteristics

Among 43,952 people initiating dialysis for ESKD in Georgia (GA), North Carolina (NC), or South Carolina (SC) between 2012–2017, 42.6% were referred within one year (Fig. 1). Overall, patients who were (vs. not) referred within 1-year of dialysis initiation were more likely to be younger, male, non-Hispanic Black, have employer-based insurance, glomerulonephritis as primary cause of ESKD, on peritoneal dialysis, did have pre-ESKD nephrology care, and fewer comorbidities, Table 1.

**Table 1:** Characteristics at dialysis initiation (2012–2017), by referral status and likelihood of being waitlisted

		All patients (*N* = 43,952)		Patients with a High Probability of Waitlisting* (*N* = 15,458)	
	Total (*N* = 43,952)	Not Referred Within 1 Year (*N* = 25,227)	Referred Within 1 Year (*N* = 18,725)	Not Referred Within 1 Year (*N* = 6,399)	Referred Within 1 Year (*N* = 9,059)
**Age**					
Median [IQR]	61.0 [51.0, 69.0]	65.0 [55.0, 72.0]	56.0 [45.0, 64.0]	56.0 [46.0–63.0]	51.0 [41.0–60.]
**Sex**					
Female	19,700 (44.8)	11,882 (47.1)	7,818 (41.8)	2,728 (42.63)	3,761 (41.52)
Male	24,252 (55.2)	13,345 (52.9)	10,907 (58.2)	3,671 (57.37)	5,298 (58.48)
**Race/ethnicity**					
Non-Hispanic White	17,660 (40.2)	11,026 (43.7)	6,634 (35.4)	2,118 (33.1)	2,955 (32.62)
Non-Hispanic Black	24,233 (55.1)	13,077 (51.8)	11,156 (59.6)	3,742 (58.48)	5,539 (61.14)
Hispanic	1,155 (2.6)	677 (2.7)	478 (2.6)	386 (6.03)	296 (3.27)
Other	904 (2.1)	447 (1.8)	457 (2.4)	153 (2.39)	269 (2.97)
**Insurance coverage**					
Medicaid	10,516 (23.9)	6,577 (26.1)	3,939 (21.0)	1,796 (28.07)	1,826 (20.16)
Medicare	17,559 (40.0)	11,691 (46.3)	5,868 (31.3)	1,804 (28.19)	1,924 (21.24)
Employer	8,623 (19.6)	3,219 (12.8)	5,404 (28.9)	1,101 (17.21)	3,222 (35.57)
Other	2,961 (6.7)	1,526 (6.1)	1,435 (7.7)	516 (8.06)	803 (8.86)
None	4,293 (9.8)	2,214 (8.8)	2,079 (11.1)	1,182 (18.47)	1,284 (14.17)
**Attributed cause of ESKD**					
Diabetes	19,943 (46.2)	11,704 (47.3)	8,239 (44.6)	1,953 (31.22)	2,825 (31.7)
Hypertension	15,663 (36.3)	8,966 (36.2)	6,697 (36.3)	2,292 (36.64)	3,253 (36.5)
Glomerulonephritis	3,211 (7.43)	1,334 (5.4)	1,877 (10.2)	864 (13.81)	1,639 (18.39)
Other	4,379 (10.1)	2,732 (11.0)	1,647 (8.9)	1,147 (18.33)	1,196 (13.42)
**Dialysis Type**					
Hemodialysis	38,968 (88.8)	23,633 (93.7)	15,335 (82.1%)	5,909 (92.37)	6,992 (77.41)
CAPD	2,173 (5.0)	672 (2.7)	1,501 (8.03%)	212 (3.31)	913 (10.11)
CCPD	2,744 (6.3)	899 (3.7)	1,845 (9.87%)	275 (4.3)	1123 (12.43)
Other	18 (0.04)	11 (0.04)	NR	1 (0.02)	5 (0.06)
** Pre-ESKD Nephrology Care**	29,002 (66.0)	15,777 (62.5)	13,225 (70.6)	3476 (63.77)	6,165 (76.08)
**Patient-Level Comorbidities**					
Diabetes	26,248 (59.7)	15,561 (61.7)	10,687 (57.1%)	2,791 (43.62)	3,789 (41.83)
Hypertension	39,399 (89.6)	22,386 (88.7)	17.013 (90.9%)	5,710 (89.23)	8,310 (91.73)
Congestive heart failure	11,553 (26.3)	7,871 (31.2)	3,682 (19.7%)	181 (2.83)	188 (2.08)
Obese (BMI > 35 kg/m^2^)	10,973 (25.1)	6,319 (25.2)	4,654 (25.0%)	541 (8.45)	842 (9.29)
Other cardiac disease	7,411 (16.9)	5,111 (20.3%)	2,300 (12.3)	472 (7.38)	490 (5.41)
Atherosclerotic heart disease	4,119 (9.4)	2,902 (11.5)	1,217 (6.5)	139 (2.17)	179 (1.98)
Peripheral vascular disease	3,702 (8.4)	2,596 (10.3)	1,106 (5.9)	47 (0.73)	39 (0.43)
Cerebrovascular accident	3,930 (8.9)	2,792 (11.1)	1,138 (6.1)	87 (1.36)	77 (0.85)
COPD	3,759 (8.6)	2,887 (11.4)	872 (4.7)	NR	NR
Cancer	2,550 (5.8)	1,931 (7.7)	619 (3.3)	390 (6.09)	185 (2.04)
Tobacco Use	3,826 (8.7)	2,411 (9.6)	1,415 (7.6)	36 (0.56)	70 (0.77)
No comorbid conditions reported	746 (1.70)	328 (1.3)	418 (2.2)	234 (3.66)	357 (3.94)

*Abbreviations: BMI* body mass index, *CAPD* continuous ambulatory peritoneal dialysis, *CCPD* continuous cycling peritoneal dialysis, *COPD* chronic obstructive pulmonary disease, *ESKD* end-stage kidney disease, *IQR* interquartile range, *NR* Not Reported, cell count < 11

^*^Defined as a propensity score of at least 0.34

### Propensity Scores (PS) for likelihood of waitlisting

**Fig. 2:**
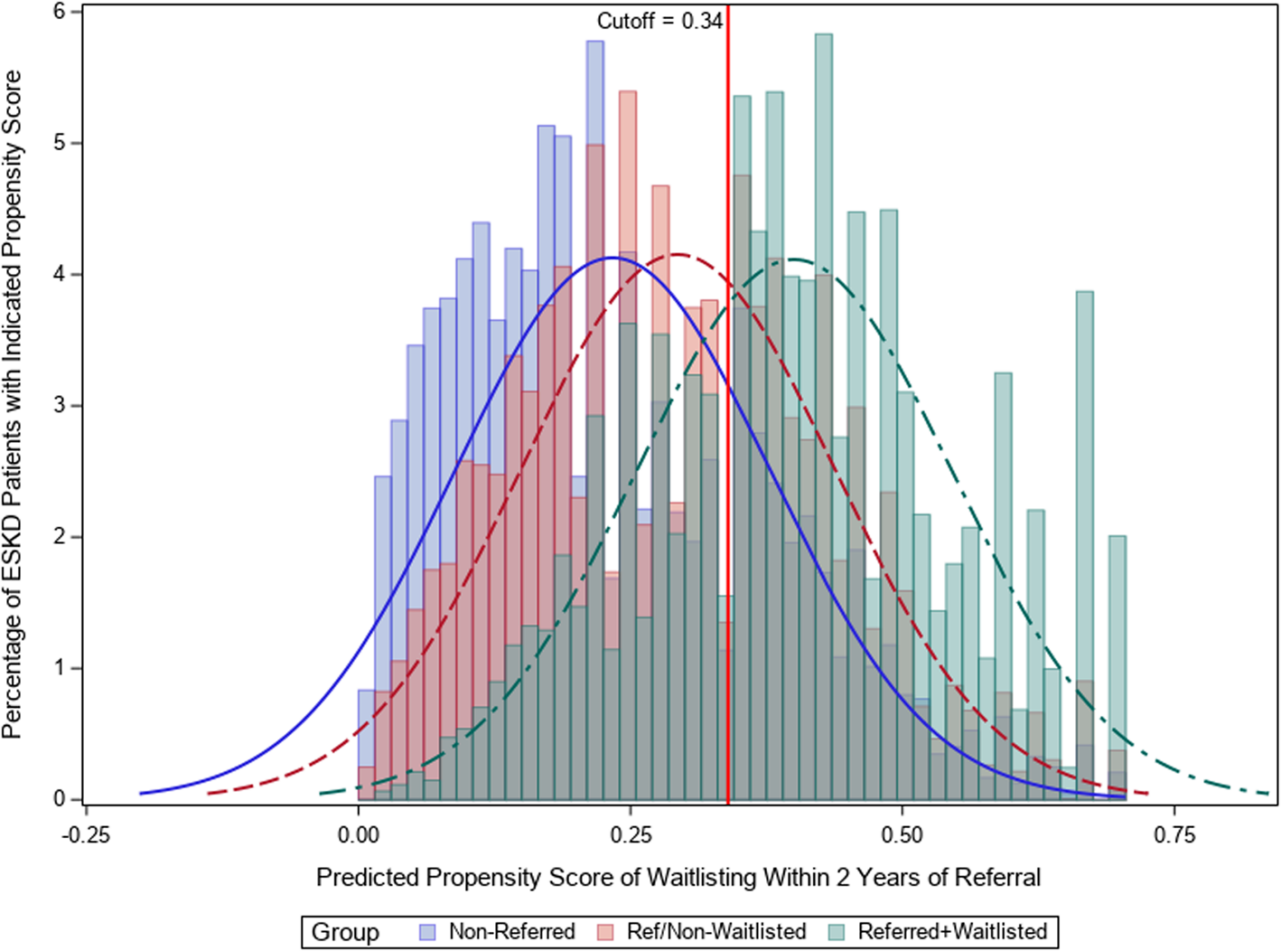
Distribution of propensity scores in full cohort

Among patients referred within 1-year, 32.9% were then waitlisted within 2 years of referral. The median propensity score (PS) was higher among waitlisted vs. non-waitlisted patients: 0.40 [IQR 0.30 –0.49] vs. 0.28 [IQR 0.18–0.40], respectively (Fig. 2). Applying the same PS (developed among referred patients) yielded a median PS of 0.21 [IQR 0.12–0.34] among non-referred patients.

### ESKD patients with a high predicted probability of waitlisting

**Fig. 3:**
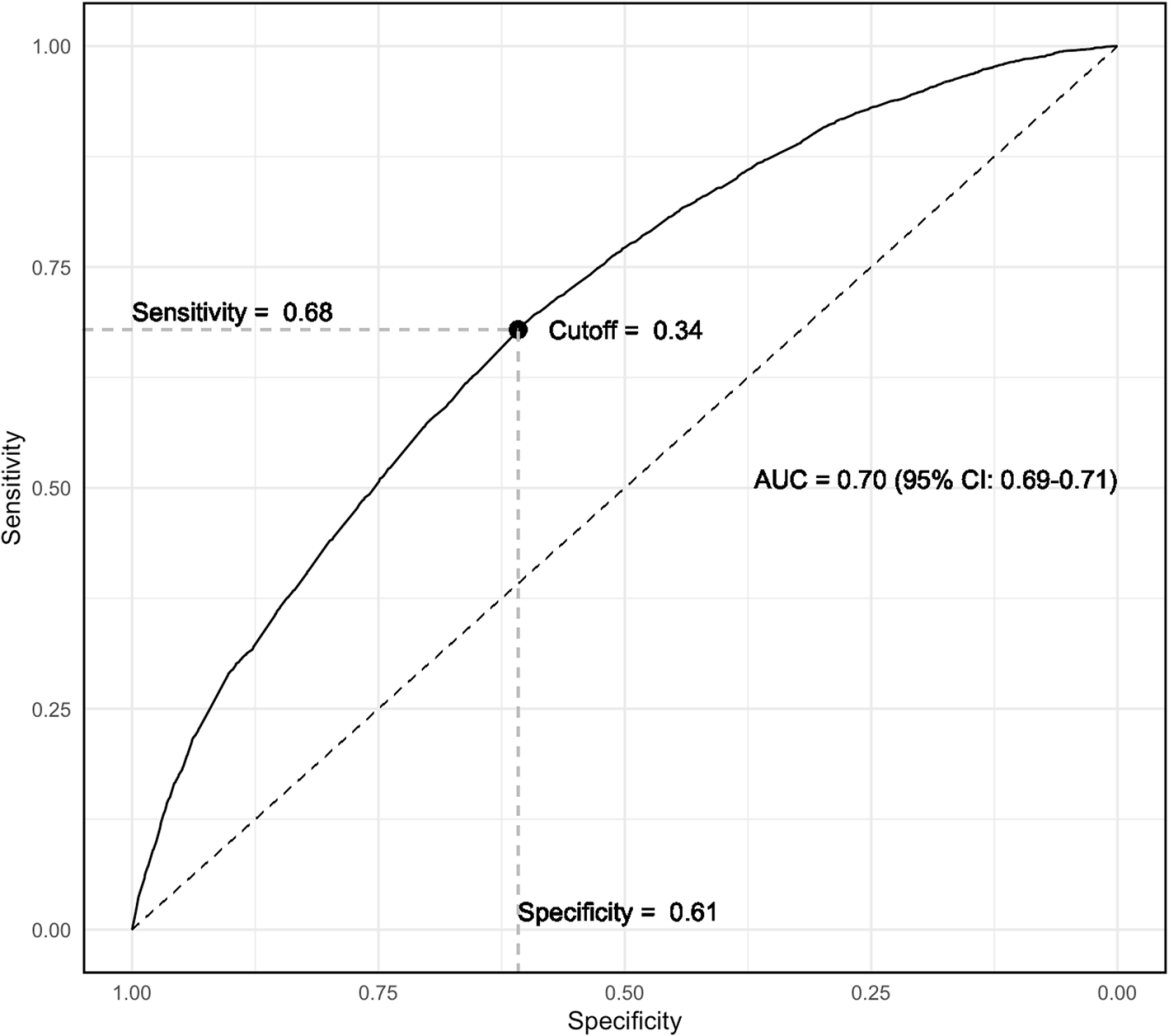
The relationship between the AUROC, sensitivity and specificity, and subsequent determination of PS-cut off using the top left corner of the ROC curve

Our model had reasonably good discrimination for identifying good transplant candidates (i.e. high predicted probability of waitlisting) with an AUROC of 0.70 (95%CI 0.69–0.71), sensitivity of 0.68 and specificity of 0.61 using a PS cut-off of 0.34 (Fig. 3). Among referred patients, this cutoff yielded positive and negative predictive values for waitlisting of 46% and 79%, respectively. Overall, we identified 15,458 individuals with a high predicted probability of waitlisting (*i.e.,* ‘good’ transplant candidates) of whom 41.4% were *not* referred within 1-year of dialysis initiation (Table 1). Non-referred (vs. referred) patients with a high predicted probability of waitlisting were more likely to be older, Hispanic, Medicaid insured, have ESKD attributed to ‘other’ causes, be on hemodialysis, not had pre-ESKD nephrology care, have cancer and other cardiac diseases (Table 1). Other comorbidities were similar in referred vs. non-referred patients, or higher in the referred population.

Among all non-referred patients, 25.4% (*n* = 6,399) were deemed to have a high predicted probability of waitlisting. Adding these patients to the existing 18,725 referred patients would increase the proportion of incident ESKD patients being referred within one year from 42.6% to 57.2% (a ~ 14.6% increase).

### Sensitivity analysis

When the PS was developed among all dialysis patients, the median PS was higher among waitlisted vs. non-waitlisted patients: 0.24 [IQR 0.16–0.35] vs. 0.10 [IQR 0.04–0.20], respectively (Figure S2). Applying the same PS (developed among all dialysis patients) yielded a median PS of 0.07 [IQR 0.03–0.18] among non-referred patients.

Our model had good discrimination for identifying good transplant candidates (i.e. high predicted probability of waitlisting) with an AUROC of 0.77 (95%CI 0.76–0.77), sensitivity of 0.71 and specificity of 0.69 using a PS cut-off of 0.18 (Figure S3). Overall, we identified 16,218 individuals with a high predicted probability of waitlisting (*i.e.,* ‘good’ transplant candidates) within 3-years of dialysis initiation of whom 26.9% were *never* referred. Non-referred (vs. referred with or without waitlisting) patients with a high predicted probability of waitlisting were more likely to be older, female, Hispanic, Medicaid insured, have ESKD attributed to ‘other’ causes, be on hemodialysis, had pre-ESKD nephrology care, and have most comorbidities (Table S2).

Among all non-referred patients, 21.1% (*n* = 4,360) were deemed to have a high predicted probability of waitlisting. Adding these patients to the existing 18,725 referred patients (from primary analysis) would increase the proportion of incident ESKD patients being referred within one year from 42.6% to 52.5% (a ~ 9.9% increase).

## Discussion

In this study, we show that at least 21–25% of ESKD patients initiating dialysis in the Southeast between 2012 and 2017 might have been good candidates for a kidney transplant but were not referred. Importantly, socioeconomic factors such as race, sex, and insurance appeared to, at least in part, explain this non-referral. As there is currently no current ‘gold standard’ for transplant candidacy, results from this study can be used to inform the setting of reasonable goals for transplant center referral among dialysis facilities. More specifically, based on results of this study, a goal of ~ 52–57% of all dialysis patients being referred for transplant may be a quality metric facilities could strive for to ensure all potentially eligible transplant candidates have the opportunity to access lifesaving treatment.

In the Southeast, if we referred those non-referred patients we believe are likely to be good transplant candidates, we would increase the proportion of referrals by ~ 10–14%, from 42.6% to 52.5–57.2%. If this increase were translated nationally, it could amount to 12,000–19,000 [6] new referrals of potentially eligible transplant candidates each year. Critics will inevitably argue that an increase in the number of referrals would simply increase the workload of transplant programs, while the supply of kidney transplants is unlikely to grow proportionately. It is also possible that an increase in total referrals (and subsequent waitlisting) may increase overall waiting times for transplant. However, we argue that a 10–14% increase in the proportion of referrals is both a meaningful and reasonable goal that would improve equity for transplant candidates, with minimal impact on efficiency of the transplant care process. Existing and extreme variation in referral patterns by dialysis facilities suggests that referral may be the right target for quality improvement [9]. Further, an increase in referrals may lead to increased awareness of the need for living organ donation, as well as an increase the pool of potential living donors (e.g., family member’s friends) associated with the referred patients.

Our findings that non-referred ‘good’ transplant candidates are more likely to be Medicaid insured and Hispanic (and female in sensitivity analyses) confirms what has been shown in other studies [9–13]. Importantly, these are factors that should not be associated with the likelihood of referral, but invariably are owing to a complex interplay between racism, sexism, and upstream social determinants of health that may impact transplant access including barriers related to transportation, health literacy, and medical mistrust [14]. Our findings highlight the need for national policies to alleviate socioeconomic barriers to referral to ensure that all potentially good candidates are referred, regardless of race, sex, or insurance status. For example, providing patients with information on pre-transplant factors (*e.g.,* waitlisting practices) rather than just post-transplant factors (*e.g.,* survival), may help patients choose a center that better fits their needs. Current quality metrics are not well aligned between dialysis facilities and transplant centers. For example, dialysis facilities aim to increase the number of patients waitlisted, while transplant centers are focused on improved post-transplant outcomes. Implementing quality metrics specific to referral may enhance both quality and equity in transplant access and ensure better alignment between dialysis facilities and transplant centers [15, 16]. However, for such quality metrics to be implemented, collection of transplant referral and evaluation data nationally will be essential. Though collection of this data alone will not lead to increased transplant access or equity, it will help us identify inequities at specific transplant care steps and develop interventions to address them [16]. Feasibility of this data collection has already been demonstrated with our E-STAR, [1] and in February 2024, HRSA (Health Resources and Services Administration) announced a directive to expand OPTN’s (Organ Procurement and Transplantation Network) data collection to include referral and evaluation. Though it may take a few years to implement at a national level, this directive demonstrates HRSAs commitment to addressing equity in transplant access and will allow us to assess national trends in referral and impact on subsequent waitlisting in the near future [17]. Finally, addressing provider bias through unconscious bias training may also be prioritized.

This study reports on the only multi-regional data that captures referral data among incident dialysis patients, and thus is uniquely positioned to answer the question ‘what proportion of potentially eligible patients are not being referred for transplant’. However, this study also has several limitations. First, the analysis was restricted to GA, NC, and SC, limiting generalizability of the results outside of the Southeast US. Recent data suggest racial disparities in waitlisting are lower in the Southeast compared to other regions where there is a disproportionate number of Black ESKD patients, and where Black patients are more likely to be referred but less likely to be evaluated as compared with White ESKD patients [18]. Given this, it is possible that other regions may have a greater or lesser proportion of non-referrals that are good transplant candidates based on race. Second, patients who may have initiated dialysis in the region but were referred to transplant centers outside of GA, NC, and SC were excluded from the study population. However, based on previous literature, we expect this to be a small proportion (i.e., < 10%) [13]. Further, a small portion of referrals (~ 7%, see Fig. 1) could not be linked to USRDS and we believe these represent referrals among late-stage chronic kidney disease (CKD) patients. Results of our study are thus applicable to people with established ESKD only. Third, we selected a PS cutoff based on the AUROC value that corresponded to the highest sensitivity and specificity. This was a relatively conservative approach to maximize the likelihood that candidates above this cutoff were medically eligible. In reality, we believe it is likely that a larger portion of non-referred patients may be appropriate candidates for transplant. However, owing to limitations of the data captured in USRDS, including a lack of data on severity of comorbidities detailed at time of dialysis initiation, we are unable to discern if adults were truly medically eligible for transplant. Fourth, the development of our PS relied on data that is captured in electronic medical records in USRDS and does not include unmeasured factors that may have influenced a clinician’s decision to refer, including transplant center waitlisting practices, patient adherence and compliance, social support, cognitive dysfunction, or illicit drug use. However, whether these factors in and of themselves should rule out patients for transplant referral remains controversial. We show that non-medical factors such as race, sex, and insurance, factors heavily tied to other social determinants of health, are associated with non-referral among otherwise eligible individuals, and these are factors known to bias a physician’s decision to refer (or not) [19, 20]. Finally, it is likely that the proportion of non-referred ‘good transplant candidates’ differs by dialysis facility. Indeed, our prior work in GA [9] showed the proportion of patients referred varied from 0 to 75% across 308 dialysis facilities. Though examining this variation will be an important direction for future investigations, the goal of our manuscript was to set a reasonable goal for referral of good transplant candidates across all dialysis facilities, with the overall goal of minimizing future variation between facilities.

## Conclusion

In this study, we show that approximately 21–25% of patients not referred for transplant may have been potentially good transplant candidates. A 10–14% increase in the proportion of patients being referred from dialysis facilities is both a meaningful and realistic goal and could lead to more qualified patients being referred and subsequently waitlisted for a lifesaving transplant.

### Supplementary Information

Supplementary Material 1. Table S1 STROBE Statement—Checklist of items that should be included in reports of *cohort studies. *Table S2: ESKD Patient Characteristics at Dialysis Initiation (2012-2017), by Referral Status, in Sensitivity Analyses (using all dialysis patients to determine PS). Figure S1 Cumulative incidence of waitlisting and death since time of referral; data used to determine appropriate cut-offs for waitlisting (i.e., 2-years). Figure S2 Distribution of propensity scores developed in full cohort and later applied to the non-referred cohort. Figure S3 The relationship between the AUC, sensitivity and specificity, and subsequent determination of PS-cut off using the top left corner of the ROC curve.

## Data Availability

The data that support the findings of this study are available upon request, but restrictions apply to the availability of these data, which were used under license for the current study, and so are not publicly available. For data requests, please contact Dr. Rachel Patzer, Regenstrief Institute.

## Abbreviations

BMI: Body Mass Index
CAPD: Continuous Ambulatory Peritoneal Dialysis
CEPD: Continuous Cycling Peritoneal Dialysis
CKD: Chronic Kidney Disease
CMS: Centers for Medicare and Medicaid
COPD: Chronic Obstructive Pulmonary Disease
ESRD: End-Stage Renal Disease
E-STAR: Early Steps to Transplant Access Registry
GA: Georgia
IQR: Interquartile Range
KRT: Kidney Replacement Therapy
NC: North Carolina
PS: Propensity Score
SC: South Carolina
USRDS: United States Renal Data System
