# Supplementary material for: Setting reasonable goals for kidney transplant referral among dialysis facilities
Source: BMC Nephrol. 2024 Jul 24;25:235. doi: 10.1186/s12882-024-03671-2 (PMC11270779; doi:10.1186/s12882-024-03671-2)
Supplement: Supplementary file 1 — Supplementary Material 1. Table S1 STROBE Statement—Checklist of items that should be included in reports of cohort studies. Table S2: ESKD Patient Characteristics at Dialysis Initiation (2012-2017), by Referral Status, in Sensitivity Analyses (using all dialysis patients to determine PS). Figure S1 Cumulative incidence of waitlisting and death since time of referral; data used to determine appropriate cut-offs for waitlisting (i.e., 2-years). Figure S2 Distribution of propensity scores developed in full cohort and later applied to the non-referred cohort. Figure S3 The relationship between the AUC, sensitivity and specificity, and subsequent determination of PS-cut off using the top left corner of the ROC curve. [file 12882_2024_3671_MOESM1_ESM.docx]

**Supplementary Material**

| **Table S1** STROBE Statement—Checklist of items that should be included in reports of *cohort studies* | | | |
| --- | --- | --- | --- |
|  | **Item No** | **Recommendation** | **Page** |
| **Title and abstract** | 1 | (*a*) Indicate the study’s design with a commonly used term in the title or the abstract | 1 |
|  |  | (*b*) Provide in the abstract an informative and balanced summary of what was done and what was found | 3-4 |
| **Introduction** | | |  |
| Background/rationale | 2 | Explain the scientific background and rationale for the investigation being reported | 5 |
| Objectives | 3 | State specific objectives, including any prespecified hypotheses | 5 |
| **Methods** | | |  |
| Study design | 4 | Present key elements of study design early in the paper | 6 |
| Setting | 5 | Describe the setting, locations, and relevant dates, including periods of recruitment, exposure, follow-up, and data collection | 6 |
| Participants | 6 | (*a*) Give the eligibility criteria, and the sources and methods of selection of participants. Describe methods of follow-up | 6 |
|  |  | (*b*) For matched studies, give matching criteria and number of exposed and unexposed |  |
| Variables | 7 | Clearly define all outcomes, exposures, predictors, potential confounders, and effect modifiers. Give diagnostic criteria, if applicable | 6-7, Suppl |
| Data sources/ measurement | 8* | For each variable of interest, give sources of data and details of methods of assessment (measurement). Describe comparability of assessment methods if there is more than one group | *6-7* |
| Bias | 9 | Describe any efforts to address potential sources of bias | 8-9 |
| Study size | 10 | Explain how the study size was arrived at | 6 |
| Quantitative variables | 11 | Explain how quantitative variables were handled in the analyses. If applicable, describe which groupings were chosen and why | 8-9 |
| Statistical methods | 12 | (*a*) Describe all statistical methods, including those used to control for confounding | 8-9 |
|  |  | (*b*) Describe any methods used to examine subgroups and interactions | 8-9 |
|  |  | (*c*) Explain how missing data were addressed | 6-9, Suppl |
|  |  | (*d*) If applicable, explain how loss to follow-up was addressed | n/a |
|  |  | (*e*) Describe any sensitivity analyses | 8-9 |
| **Results** | | |  |
| Participants | 13* | (a) Report numbers of individuals at each stage of study—eg numbers potentially eligible, examined for eligibility, confirmed eligible, included in the study, completing follow-up, and analysed | 9, Fig 1 |
|  |  | (b) Give reasons for non-participation at each stage | Fig 1 |
|  |  | (c) Consider use of a flow diagram | Fig 1 |
| Descriptive data | 14* | (a) Give characteristics of study participants (eg demographic, clinical, social) and information on exposures and potential confounders | 9, Table 1 & Suppl T2 |
|  |  | (b) Indicate number of participants with missing data for each variable of interest | Table 1 |
|  |  | (c) Summarise follow-up time (eg, average and total amount) | 10 |
| Outcome data | 15* | Report numbers of outcome events or summary measures over time | Suppl 2 |
| Main results | 16 | (*a*) Give unadjusted estimates and, if applicable, confounder-adjusted estimates and their precision (eg, 95% confidence interval). Make clear which confounders were adjusted for and why they were included | 10, Table 2 |
|  |  | (*b*) Report category boundaries when continuous variables were categorized | n/a |
|  |  | (*c*) If relevant, consider translating estimates of relative risk into absolute risk for a meaningful time period | n/a |
| Other analyses | 17 | Report other analyses done—eg analyses of subgroups and interactions, and sensitivity analyses | 11, Figure 2 |
| **Discussion** | | |  |
| Key results | 18 | Summarise key results with reference to study objectives | 11-12 |
| Limitations | 19 | Discuss limitations of the study, taking into account sources of potential bias or imprecision. Discuss both direction and magnitude of any potential bias | 15 |
| Interpretation | 20 | Give a cautious overall interpretation of results considering objectives, limitations, multiplicity of analyses, results from similar studies, and other relevant evidence | 15-16 |
| Generalisability | 21 | Discuss the generalisability (external validity) of the study results | 11-15 |
| **Other information** | | |  |
| Funding | 22 | Give the source of funding and the role of the funders for the present study and, if applicable, for the original study on which the present article is based | 17 |

| **Table S2:** ESKD Patient Characteristics at Dialysis Initiation (2012-2017), by Referral Status, in Sensitivity Analyses (using all dialysis patients to determine PS) | | | | | | |
| --- | --- | --- | --- | --- | --- | --- |
|  | **All ESKD patients**  **(N=43,952)** | | | **ESKD Patients with a High Probability of Waitlisting* (N=16,218)** | | |
|  | **Not Referred  (N=20,674)** | **Referred + not waitlisted (N=16,647)** | **Referred + waitlisted (N=6,631)** | **Not Referred N=4,360** | **Referred + not waitlisted N=7,183** | **Referred + waitlisted N=4,675** |
| **Age** |  |  |  |  |  |  |
| Median [IQR] | 64 [58, 73] | 55 [47, 65] | 53 [42, 62] | 56 [46, 63] | 50 [41, 59] | 48 [39, 58] |
| **Sex** |  |  |  |  |  |  |
| Female | 9,958 (48.17) | 7,112 (42.72) | 2,630 (39.66) | 1,934 (44.36) | 3,015 (41.97) | 1,902 (40.68) |
| **Race/ethnicity** |  |  |  |  |  |  |
| Non-Hispanic White | 9,726 (47.04) | 5,521 (33.17) | 2,413 (36.39) | 1,523 (34.93) | 1,929 (26.86) | 1,623 (34.72) |
| Non-Hispanic Black | 10,042 (48.57) | 10,440 (62.71) | 3,751 (56.57) | 2,432 (55.78) | 4,897 (68.17) | 2,688 (57.5) |
| Hispanic | 548 (2.65) | 371 (2.23) | 236 (3.56) | 297 (6.81) | 212 (2.95) | 192 (4.11) |
| Other | 358 (1.73) | 315 (1.89) | 231 (3.48) | 108 (2.48) | 145 (2.02) | 172 (3.68) |
| **Insurance coverage** |  |  |  |  |  |  |
| Medicaid | 5,352 (25.89) | 4,233 (25.43) | 931 (14.04) | 1,325 (30.39) | 1,982 (27.59) | 676 (14.46) |
| Medicare | 10,266 (49.66) | 5,787 (34.76) | 1,506 (22.71) | 1,243 (28.51) | 1,594 (22.19) | 755 (16.15) |
| Employer | 2,385 (11.54) | 3,408 (20.47) | 2,830 (42.68) | 649 (14.89) | 1,784 (24.84) | 2,165 (46.31) |
| Other | 1,183 (5.72) | 1,158 (6.96) | 620 (9.35) | 343 (7.87) | 549 (7.64) | 478 (10.22) |
| None | 1,488 (7.20) | 2,061 (12.38) | 744 (11.22) | 800 (18.35) | 1,274 (17.74) | 601 (12.86) |
| **Attributed cause of ESKD** |  |  |  |  |  |  |
| Diabetes | 9,695 (47.88) | 7,953 (48.41) | 2,295 (35.21) | 1,512 (35.48) | 2,665 (37.6) | 1,274 (27.73) |
| Hypertension | 7,235 (35.73) | 6,061 (36.89) | 2,367 (36.31) | 1,614 (37.88) | 2,805 (39.58) | 1,689 (36.76) |
| Glomerulonephritis | 975 (4.82) | 1,163 (7.08) | 1,073 (16.46) | 470 (11.03) | 905 (12.77) | 995 (21.65) |
| Other | 2,343 (11.57) | 1,253 (7.63) | 783 (12.01) | 665 (15.61) | 712 (10.05) | 637 (13.86) |
| **Dialysis Type** |  |  |  |  |  |  |
| Hemodialysis | 19,509 (94.41) | 14,633 (87.98) | 4,826 (73.05) | 4,090 (93.81) | 6,130 (85.45) | 3,283 (70.48) |
| CAPD | 490 (2.37) | 928 (5.58) | 755 (11.43) | 121 (2.78) | 480 (6.69) | 594 (12.75) |
| CCPD | 657 (3.18) | 1065 (6.40) | 1,022 (15.47) | 149 (3.42) | 559 (7.79) | 779 (16.72) |
| Other | NR | NR | NR | NR | NR | NR |
| **Pre-ESKD Nephrology Care** | 12,899 (71.56) | 11,231 (76.02) | 4,872 (80.64) | 2,314 (62.74) | 4,503 (71.73) | 3,336 (78.75) |
| **Patient-Level Comorbidities** |  |  |  |  |  |  |
| Diabetes | 12,921 (62.5) | 10,217 (61.37) | 3,110 (46.9) | 2,111 (48.42) | 3,518 (48.98) | 1,790 (38.29) |
| Hypertension | 18258 (88.31) | 15,117 (90.81) | 6,024 (90.85) | 3,936 (90.28) | 6,670 (92.86) | 4,293 (91.83) |
| Congestive heart failure | 6,794 (32.86) | 4,030 (24.21) | 729 (10.99) | 118 (2.71) | 233 (3.24) | 92 (1.97) |
| Obese (BMI>35 kg/m^2^) | 4,962 (24.20) | 4,717 (28.48) | 1,294 (19.61) | 487 (11.17) | 1340 (18.66) | 630 (13.48) |
| Other cardiac disease | 4,501 (21.77) | 2,316 (13.91) | 594 (8.96) | 304 (6.97) | 383 (5.33) | 215 (4.6) |
| Atherosclerotic heart disease | 2,566 (12.41) | 1,282 (7.70) | 271 (4.09) | 109 (2.5) | 155 (2.16) | 83 (1.78) |
| Peripheral vascular disease | 2,310 (11.17) | 1,192 (7.16) | 200 (3.02) | 50 (1.15) | 71 (0.99) | 31 (0.66) |
| Cerebrovascular accident | 2,436 (11.78) | 1,256 (7.54) | 238 (3.59) | 50 (1.15) | 63 (0.88) | 33 (0.71) |
| COPD | 2,639 (12.76) | 1,029 (6.18) | 91 (1.37) | NR | NR | NR |
| Cancer | 1,738 (8.41) | 632 (3.80) | 180 (2.71) | 89 (2.04) | 62 (0.86) | 44 (0.94) |
| Tobacco Use | 2,012 (9.73) | 1,557 (9.35) | 257 (3.88) | 49 (1.12) | 94 (1.31) | 29 (0.62) |
| No comorbid conditions reported | 232 (1.12) | 253 (1.52) | 261 (3.94) | 144 (3.3) | 206 (2.87) | 238 (5.09) |
| *Abbreviations:* BMI = body mass index; CAPD = continuous ambulatory peritoneal dialysis; CCPD = continuous cycling peritoneal dialysis; COPD = chronic obstructive pulmonary disease; ESKD = end-stage kidney disease; IQR = interquartile range; NR = Not Reported, cell count <11 | | | | | | |

**Figure S1** Cumulative incidence of waitlisting and death since time of referral; data used to determine appropriate cut-offs for waitlisting (i.e., 2-years


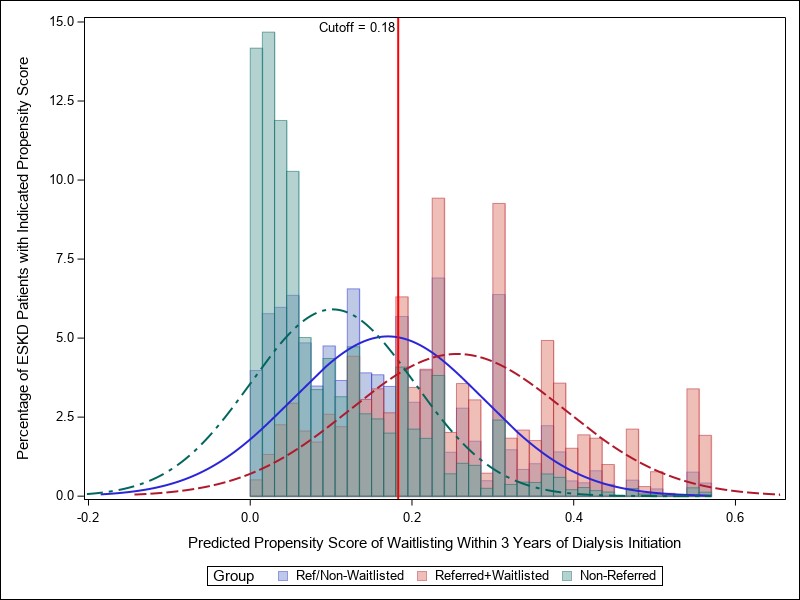


**Figure S2** Distribution of propensity scores developed in full cohort and later applied to the non-referred cohort


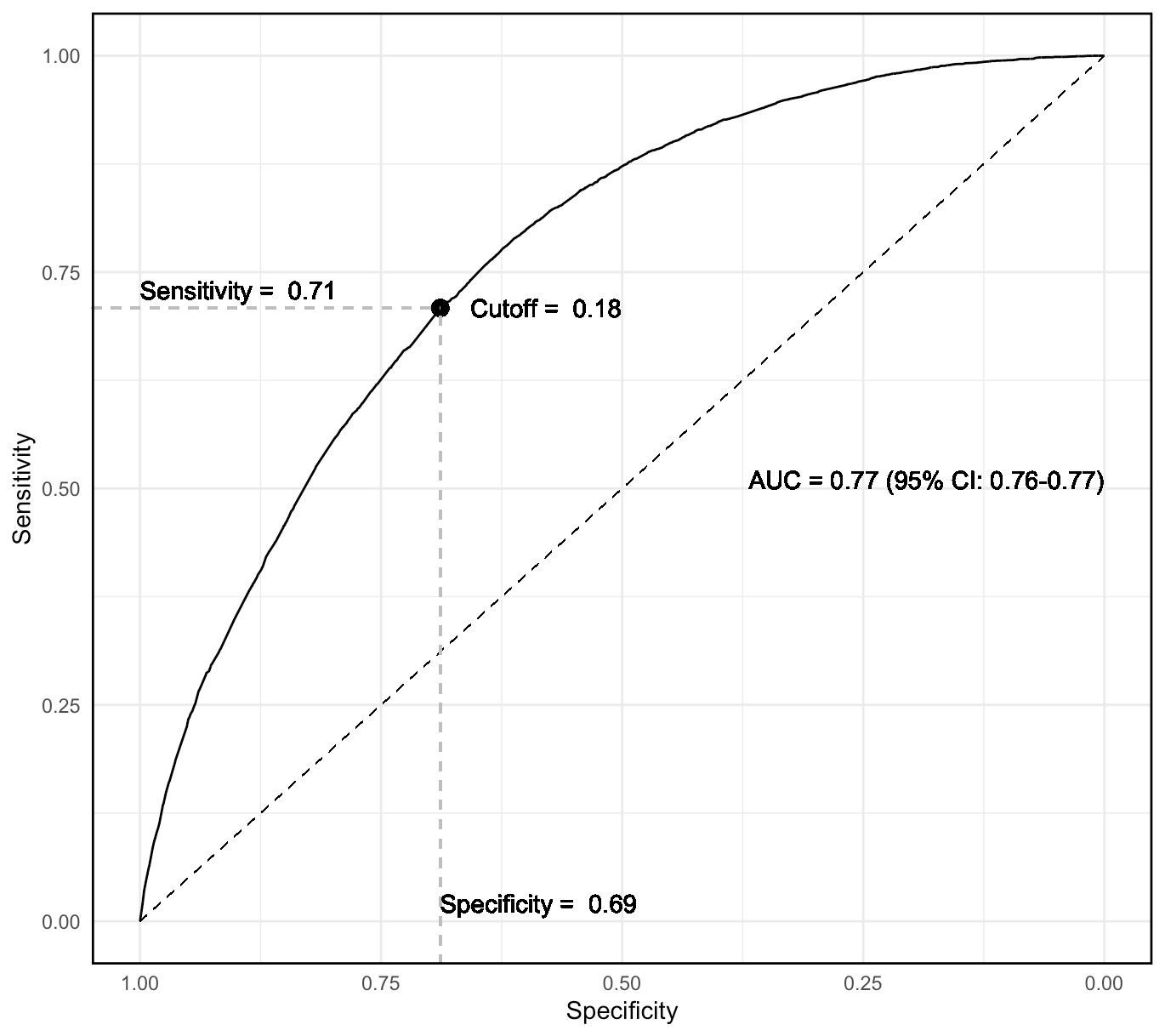


**Figure S3** The relationship between the AUC, sensitivity and specificity, and subsequent determination of PS-cut off using the top left corner of the ROC curve

**Figure S3** Distribution of propensity scores in full cohort
